# Nanostructured Magnetic Particles for Removing Cyanotoxins: Assessing Effectiveness and Toxicity In Vitro

**DOI:** 10.3390/toxins16060269

**Published:** 2024-06-13

**Authors:** Alejandro Cao, Natalia Vilariño, Lisandra de Castro-Alves, Yolanda Piñeiro, José Rivas, Ana M. Botana, Cristina Carrera, María J. Sainz, Luis M. Botana

**Affiliations:** 1 Departamento de Farmacología, Farmacia y Tecnología Farmacéutica, Facultad de Veterinaria, Universidad de Santiago de Compostela, 27002 Lugo, Spain; alejandro.cao.cancelas@usc.es (A.C.); cristina.carrera@usc.es (C.C.); luis.botana@usc.es (L.M.B.); 2 Departamento de Física Aplicada, Facultad de Física, Universidad de Santiago de Compostela, 15782 Santiago de Compostela, Spain; lisandracristina.decastro@usc.es (L.d.C.-A.); y.pineiro.redondo@usc.es (Y.P.); 3 Instituto de Materiales iMATUS, Universidad de Santiago de Compostela, 15782 Santiago de Compostela, Spain; 4 Instituto de Investigación Sanitaria (IDIS), Universidad de Santiago de Compostela, 15782 Santiago de Compostela, Spain; 5 Departamento de Química Analítica, Nutrición y Bromatología, Facultad de Ciencias, Universidad de Santiago de Compostela, 27002 Lugo, Spain; anamaria.botana@usc.es; 6 Hospital Veterinario Universitario Rof Codina, Universidad de Santiago de Compostela, 27002 Lugo, Spain; 7 Departamento de Producción Vegetal y Proyectos de Ingeniería, Facultad de Veterinaria, Universidad de Santiago de Compostela, 27002 Lugo, Spain; mj.sainz@usc.es

**Keywords:** magnetic nanoparticles, cyanotoxins removal, drinking water safety

## Abstract

The rise in cyanobacterial blooms due to eutrophication and climate change has increased cyanotoxin presence in water. Most current water treatment plants do not effectively remove these toxins, posing a potential risk to public health. This study introduces a water treatment approach using nanostructured beads containing magnetic nanoparticles (MNPs) for easy removal from liquid suspension, coated with different adsorbent materials to eliminate cyanotoxins. Thirteen particle types were produced using activated carbon, CMK-3 mesoporous carbon, graphene, chitosan, 2,2,6,6-tetramethylpiperidine-1-oxyl (TEMPO)-oxidised cellulose nanofibers (TOCNF), esterified pectin, and calcined lignin as an adsorbent component. The particles’ effectiveness for detoxification of microcystin-LR (MC-LR), cylindrospermopsin (CYN), and anatoxin-A (ATX-A) was assessed in an aqueous solution. Two particle compositions presented the best adsorption characteristics for the most common cyanotoxins. In the conditions tested, mesoporous carbon nanostructured particles, P1-CMK3, provide good removal of MC-LR and Merck-activated carbon nanostructured particles, P9-MAC, can remove ATX-A and CYN with high and fair efficacy, respectively. Additionally, in vitro toxicity of water treated with each particle type was evaluated in cultured cell lines, revealing no alteration of viability in human renal, neuronal, hepatic, and intestinal cells. Although further research is needed to fully characterise this new water treatment approach, it appears to be a safe, practical, and effective method for eliminating cyanotoxins from water.

## 1. Introduction

The natural environment contains substances that can pose risks to the health and general well-being of humans and animals. Cyanotoxins deserve special mention due to their well-documented toxicity [1] and the increasing occurrences of poisoning in both animal and human populations [2,3]. Cyanotoxins are compounds synthesised by cyanobacteria, a phylum of photosynthetic prokaryotes that can be found in diverse environments including fresh, brackish, or marine waters, sunlight-exposed surfaces of rocks, and soils [4,5]. Cyanotoxins are predominantly found in the intracellular space of cyanobacteria. The exact biological purpose of these molecules remains unclear. Some hypotheses suggest that they might function as a defense mechanism against grazing or competition, while other authors suggest that they may play a role in the physiological functions of cyanobacteria [6].

Cyanotoxin production occurs during the proliferation of specific cyanobacterial species. Cyanobacterial blooms are rapid and substantial increases in cyanobacterial biomass within a short timeframe, often accompanied by a reduction in phytoplankton diversity [7]. Such blooms can result in extensive coverage of the water surface, sometimes changing the water’s colour [5], which is commonly termed a ‘blue-green algae bloom’. When these blooms are caused by toxin-producing cyanobacterial species, the concentration of cyanotoxins in the water can pose significant health risks to humans and animals [8]. Furthermore, some research studies have established a correlation between water eutrophication and/or climate change and an increase in the frequency of cyanobacterial blooms [9,10]. Consequently, this public health threat has prompted many countries and the World Health Organization (WHO) to actively implement legislative measures and recommendations aimed at reducing the adverse health effects of these toxins [11,12,13,14,15]. In fact, WHO has recently advocated for the comprehensive assessment of all microcystins in water sources when applying a 1 µg/L limit [13].

Over a hundred cyanotoxins with different origins, chemical structures, and characteristics have been identified. However, three toxin classes such as MC-LR, CYN, and ATX-A are particularly prevalent in freshwater blooms [16]. Evidence of the toxic effects of these substances in humans and animals has been accumulating over the years [17,18].

MC-LR acute intoxication signs in humans include liver damage, gastrointestinal upset, and fever [19]. In addition, numerous studies have investigated the long-term effects of MC-LR exposure, demonstrating its ability to induce liver, cardiovascular, and reproductive problems in mice [20,21,22,23]. The International Agency for Research on Cancer (IARC) has classified it as a potential tumour promoter [24]. In the early stages of oral intoxication with CYN, humans may experience anorexia, constipation, vomiting, headache, fever, and abdominal pain. Subsequently, renal and hepatic symptoms may intensify, leading to acidotic shock, bloody diarrhoea, and bleeding mucous membranes [25]. Symptoms of ATX-A poisoning, identified from the first confirmed human intoxication due to ingestion of ATX-A-contaminated seafood, may include gastrointestinal symptoms and neurological problems such as blurred vision, ataxia, muscle cramps, or paresthesia [17]. Evidence indicates human fatalities resulting from MC-LR intoxication [26], as well as animal deaths associated with the three cyanotoxins [27,28,29].

Current water treatment techniques focus primarily on the removal of cyanobacterial biomass, but the removal of free cyanotoxins from water is a more complex challenge [30]. Some studies support the efficacy of oxidising chemicals, which are already being used in conventional purification stations, in removing free cyanotoxins from water [31,32,33,34]. However, the generation of other harmful compounds like trihalomethanes [35] or the requirement for additional techniques such as UV radiation [36] contribute to the high cost and unsafety concerns of these treatments. Another method described in the literature is the use of bacteria capable of degrading cyanotoxins in water [37,38,39]. However, this mechanism requires long periods of time, and the introduction of these bacteria may disrupt the ecosystem [40]. These concerns make these methods not suitable for the safe and practical removal of cyanotoxins from water in treatment stations. In recent years, adsorbent materials like graphene oxide [41], organic polymers [42,43], waste biomass [44], protonated mesoporous graphitic carbon nitride [45], biochars [46], or carbon [47] have been tested. While their adsorption efficiency varies depending on the composition and size, the subsequent removal of these materials from the water proves challenging, and the remaining residues can be harmful to people.

In this study, a complementary method for toxin removal based on nanostructured, superparamagnetic particles composed of a porous biopolymeric matrix containing nano-/micro-particles is investigated. These non-aggregating macroparticles allow for practical withdrawal from liquid matrixes using magnetic forces or other physical methods. The effectiveness of cyanotoxin removal of thirteen types of nanostructured particles with different adsorbents has been explored. In addition, in vitro toxicity in cell cultures was also tested for an initial assessment of the animal and environmental safety of these materials.

## 2. Results

### 2.1. Cyanotoxin Adsorption by Thirteen Nanostructured Superparamagnetic Particle Types

Thirteen magnetic nanostructured particles with different coating materials for toxin adsorption (see Table 1) were tested for the removal of MC-LR, CYN, and ATX-A from the solution in water. For each experiment, 3 wet nanostructured particles (stored in Milli-Q water) were added to a volume of 4.5 mL of a solution of 60 μg/L MC-LR, 60 μg/L CYN or 10 μg/L ATX-A or Milli-Q water. Cyanotoxin concentrations were selected considering a moderately high contamination level of 60 µg/L of MC-LR, which can reach concentrations higher than 100 µg/L, but do not usually exceed 20 µg/L [13], and the same concentration was used for CYN. For ATX-A, owing to its smaller molecular size, the concentration was selected by matching MC-LR molar concentration. Adsorption kinetics were explored by collecting samples at different time points for 2 h. Longer exposure was not considered practical for water treatment plants. Constant shaking during the experiment ensured adequate contact of the nanostructured particles with the toxin in solution. Toxin adsorption was evaluated after quantification by UHPLC-MS/MS of the toxin concentration in water samples collected during the experiment. In addition, control samples of toxin solution in the same conditions except for the absence of particles were included in every experiment. Samples without nanostructured particles showed no loss of toxin during the assay. Adsorption is reported as the percentage of toxin that does not remain in solution after the indicated time from the addition of nanostructured particles.

**Figure 1:**
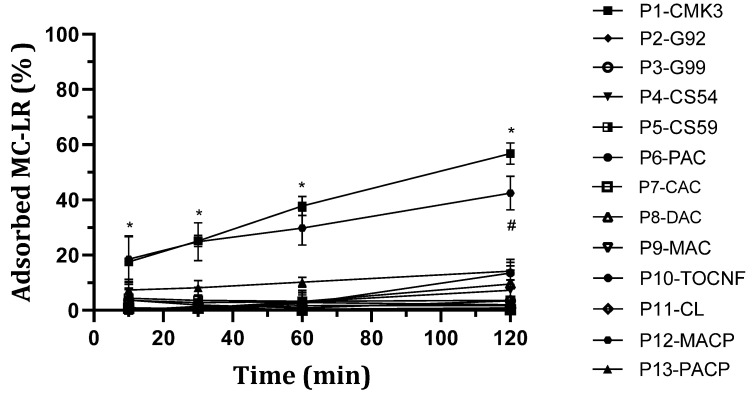
Adsorption kinetics of thirteen types of adsorbent nanostructured magnetic particles for MC-LR. For each one, three nanostructured particles were incubated for 10, 30, 60, and 120 min with 4.5 mL of 60 μg/L MC-LR. Toxin solution was prepared in Milli-Q water and the amount of toxin remaining in solution at the indicated time was quantified by LC-MS/MS. Adsorption was calculated from the amount of toxin removed from solutions versus initial concentration (mean ± SD, n = 3). * Statistically significant difference vs. P2-G92, P3-G99, P4-CS54, P5-CS59, P6-PAC, P7-CAC, P8-DAC, P9-MAC, P10-TOCNF, P11-CL, and P13-PACP. # Statistically significant difference vs. P4-CS54, P5-CS59, P2-G92, P3-G99, P7-CAC, P8-DAC, P10-TOCNF, and P11-CL (*p* < 0.05).

**Figure 2:**
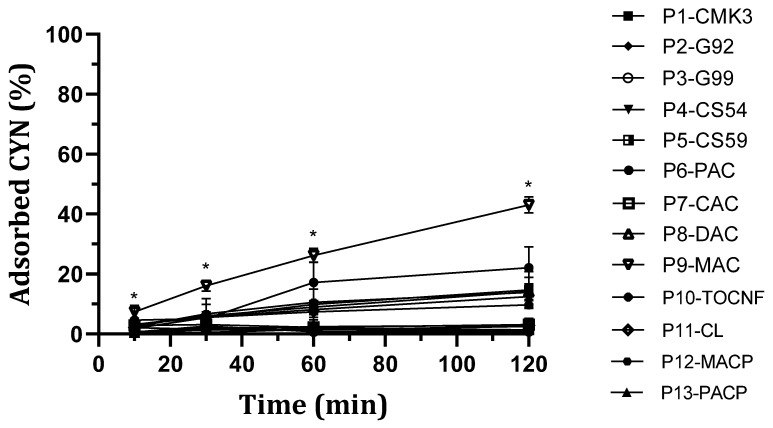
Adsorption kinetics of thirteen types of adsorbent nanostructured magnetic particles for CYN. For each one, three nanostructured particles were incubated for 10, 30, 60, and 120 min with 4.5 mL of 60 μg/L CYN. Toxin solution was prepared in Milli-Q water and the amount of toxin remaining in solution at the indicated time was quantified by LC-MS/MS. Adsorption was calculated from the amount of toxin removed from solutions versus initial concentration (mean ± SD, n = 3). * Statistically significant difference vs. P2-G92, P3-G99, P4-CS54, and P5-CS59 at time 10 min, and vs. P2-G92, P4-CS54, P5-CS59, P7-CAC, P8-DAC, P10-TOCNF, P11-CL, P12-MACP, and P13-PACP at times 30, 60, and 120 (*p* < 0.05).

**Figure 3:**
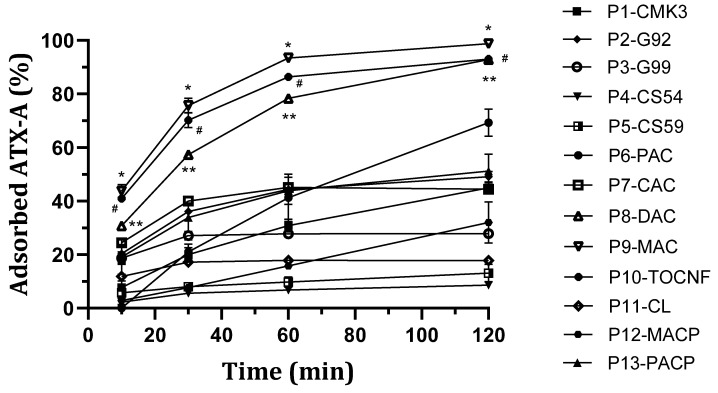
Adsorption kinetics of thirteen types of adsorbent nanostructured magnetic particles for ATX-A. For each one, three nanostructured particles were incubated for 10, 30, 60, and 120 min with 4.5 mL of 10 μg/L ATX-A. Toxin solution was prepared in Milli-Q water and the amount of toxin remaining in solution at the indicated time was quantified by LC-MS/MS. Adsorption was calculated from the amount of toxin removed from solutions versus initial concentration (mean ± SD, n = 3). * Statistically significant difference vs. P1-CMK3, P2-G92, P3-G99, P4-CS54, P5-CS59, P6-PAC, P7-CAC, P8-DAC (10 and 60 min), P10-TOCNF (60 and 120 min), P11-CL, P12-MACP, and P13-PACP. # Statistically significant difference vs. P1-CMK3, P2-G92, P3-G99, P4-CS54, P5-CS59, P6-PAC (10 and 30 min), P7-CAC, P8-DAC, P10-TOCNF (60 and 120 min), P11-CL, P12-MACP (10 and 120 min), and P13-PACP. ** Statistically significant difference vs. P1-CMK3, P2-G92 (30, 60, and 120 min), P3-G99 (30, 60, and 120 min), P4-CS54, P5-CS59 (30, 60, and 120 min), P6-PAC (10 min), P7-CAC, P10-TOCNF (60 min), P11-CL, P12-MACP (10 and 120 min), and P13-PACP (10, 30, and 60 min). Time points in brackets indicate significance only at that time. The following statistically significant differences are not indicated by symbols in the graph: P2-G92 vs. P3-G99, P4-CS54, P5-CS59, and P11-CL at 30, 60, and 120 min, and only at 30 and 60 min vs. P1-CMK3 and P12-MACP; P3-G99 vs. P4-CS54, P5-CS59 and P11-CL at 30, 60, and 120 min and P1-CMK3 (120 min), P7-CAC (30 and 120 min), and P12-MACP (30 and 60 min); and P6-PAC vs. P3-G99, P4-CS54, P5-CS59, P11-CL, and P12-MACP at 120 min (*p* < 0.05).

**Figure 4:**
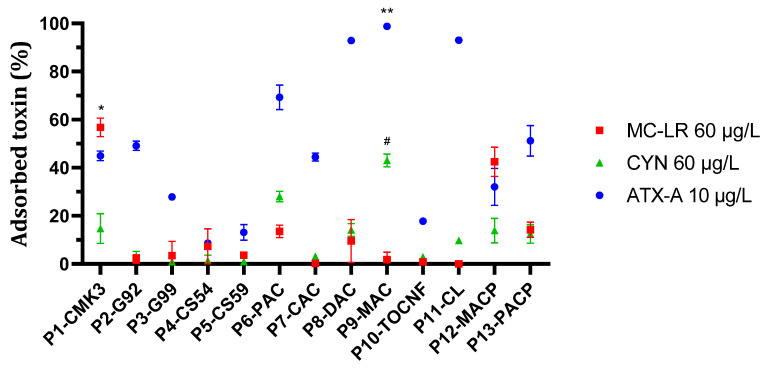
Adsorption efficiency of thirteen types of adsorbent nanostructured magnetic particles for MC-LR, CYN, and ATX-A at 120 min. Experiments were performed as in Figure 1, Figure 2 and Figure 3. Toxin removal from water at 120 min for the three toxins with all particle types tested is shown for comparison (mean ± SD, n = 3). * Statistically significant vs. P2-G92, P3-G99, P4-CS54, P5-CS59, P6-PAC, P7-CAC, P8-DAC, P9-MAC, P10-TOCNF, P11-CL, and P13-PACP. # Statistically significant vs. P2-G92, P4-CS54, P5-CS59, P7-CAC, P8-DAC, P10-TOCNF, P11-CL, P12-MACP, and P13-PACP. ** Statistically significant vs. P1-CMK3, P2-G92, P3-G99, P4-CS54, P5-CS59, P6-PAC, P7-CAC, P8-DAC, P10-TOCNF, P11-CL, P12-MACP, and P13-PACP. Symbols for statistical significance are shown only for the particles with the best performance for each toxin; for more information about statistically significant differences for other particles, see Figure 1, Figure 2 and Figure 3 (*p* < 0.05).

The results showed variations in adsorption capability depending on the nanostructured particle type and the toxin. In general, MC-LR and CYN adsorption displayed a slow kinetics, with continuous linear increase along the experiment (Figure 1 and Figure 2, respectively), while adsorption kinetics was faster for ATX-A, reaching values close to a plateau at 60 min (Figure 3). To compare adsorption efficiency among particle types for the three toxins, data at 120 min were considered (Figure 4). None of the nanostructured particles displayed the highest efficiency for the three toxins. The best adsorption results for MC-LR were obtained with P1-CMK3, whose main adsorbent material is the ordered mesoporous carbon CMK-3. These particles removed 56.76 ± 3.8% (mean ± SD) MC-LR from the 60 µg/L solution. P12-MACP nanostructured particles, coated with a mixture of pectin and activated carbon, removed 42.47 ± 6.0% of the toxin. The amount of toxin adsorbed per gram of dry nanostructured particle was calculated using average particle weight (Table 1). Actually, the particles used for these experiments were never dried, because the rewetting of previously dried nanostructured particles demonstrated worse adsorption efficiency. Therefore, weighting of the very same dry particles used in the experiment was not possible. For P1-CMK3 and P12-MACP, the rate of MC-LR removed per gram of material was 6.75 ± 0.4 and 22.48 ± 2.7 μg/g, respectively. The other tested particles did not show significant elimination rates for MC-LR.

Regarding CYN, although it was the most problematic of the three toxins in terms of adsorption efficiency, nanostructured particles P9-MAC and P6-PAC exhibited better kinetics compared to the others (Figure 2). These particles are coated with two types of activated carbon on their surfaces. Merck-activated carbon particles, P9-MAC, exhibited a toxin removal of 43.00 ± 2.7% at 120 min from a 60 µg/L CYN solution, while Panreac-activated carbon particles, P6-PAC, showed a removal percentage of 22.14 ± 6.9% in the same conditions (Figure 4). For the P9-MAC nanostructured particles, the adsorption rate demonstrates a positive correlation with elapsed time, suggesting a continual increase without apparent saturation within the observed timeframe. In contrast, the adsorption of CYN by P6-PAC nanostructured particles seemed to reach a plateau around 60 min after addition, indicating that the maximum adsorption capacity may be attained within this specific time interval. The rates of CYN removed per gram at 120 min were 8.08 ± 0.3 μg/g for P9-MAC and 6.99 ± 1.9 μg/g for P6-PAC particles. For the other particle types, the adsorption percentage remained either negligible or zero.

Concerning ATX-A removal, it was observed that, although most nanostructured particles demonstrated some degree of toxin adsorption, three materials exhibited superior performance compared to others (Figure 3). Specifically, Merck-activated carbon particles, P9-MAC, showed the best results, achieving a removal rate of 98.76 ± 0.06% for ATX-A from a 10 µg/L solution, corresponding to an adsorption rate of 3.28 ± 0.6 μg/g. TOCNF nanostructured particles, P10-TOCNF, and Desotec-activated carbon particles, P8-DAC, followed, with removals of 93.04 ± 0.8% and 92.90 ± 0.6% corresponding to the adsorption rates of 5.8 ± 1.07 μg/g and 4.12 ± 0.81 μg/g, respectively, using the same experimental conditions. Particles P9-MAC and P10-TOCNF exhibited faster kinetics, with an almost maximum adsorption level after 60 min, whereas for P8-DAC adsorption at 120 min was clearly higher than at 60 min (Figure 3). Other nanostructured particles demonstrated fair adsorption effectiveness in ATX-A adsorption. Specifically, Panreac carbon particles, P6-PAC, revealed a removal rate of 69.28 ± 5.06% or 2.61 ± 0.28 μg/g; mesoporous carbon particles, P1-CMK3, exhibited a removal rate of 44.95 ± 1.9% or 1.32 ± 0.05 μg/g; and Cabot-activated carbon nanostructured particles, P7-CAC, showed a removal rate of 44.45 ± 1.66% or 3.87 ± 0.86 μg/g. Graphene nanostructured particles had medium to low effectiveness since they showed an adsorption rate of 49.11 ± 1.9% (2.04 ± 0.1 μg/g) for P2-G92 and 27.9 ± 1.33% (0.72 ± 0.03 μg/g) for P3-G99. Chitosan and calcinated lignin demonstrated insufficient efficacy in removing any of the three most prevalent toxins.

Following the adsorption procedure, desorption experiments were conducted with three particle types, P1-CMK3, P6-PAC, and P9-MAC, using 75% acetonitrile. For these three nanostructured particles, the recovery of the three toxins ranged from 60% to 100% of the adsorbed toxin.

### 2.2. Cyanotoxin Adsorption to Nanostructured Particles in Cyanobacteria Lysate

**Figure 5:**
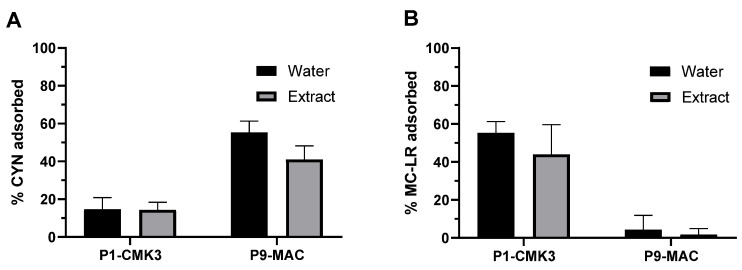
Evaluation of the adsorption efficiency of P1-CMK3 and P9-MAC particles in a cyanobacteria extract. An aqueous extract of *Aphanizomenon ovalisporum*, 1 mg/mL, was obtained by sonication, diluted (1:15), and incubated for 120 min with particles P1-CMK3 or P9-MAC (3 particles in 4.5 mL of diluted extract). A control in Milli-Q water spiked with 300 μg/L CYN and 3 μg/L MC-LR was performed simultaneously. The amount of toxin remaining in solution at 120 min was quantified by LC-MS/MS. Adsorption was calculated from the amount of toxin removed from solutions versus initial concentration for CYN (**A**) and MC-LR (**B**), both for extract and Milli-Q water control (mean ± SD, n = 3).

An extract of the cyanobacteria *Aphanizomenon ovalisporum* at a concentration of 1 mg biomass per mL of Milli-Q water was prepared by cell lysis using sonication and toxin concentration in this extract was quantified by LC-MS/MS. This extract contained 4500 μg/L and 47 μg/L of CYN and MC-LR, respectively, along with the presence of MC-LA and des-MC-LR at levels below 1 μg/L. Subsequently, particles P1-CMK3 and P9-MAC, which had the best performance in Milli-Q water in terms of adsorption ratios for MC-LR and CYN, respectively, were tested for toxin adsorption efficiency in the cyanobacteria extract. The experiments were performed as previously described with a 1:15 dilution of this aqueous extract. A control of Milli-Q water spiked with the same concentration of MC-LR and CYN as the diluted extract (3 µg/L and 300 µg/L, respectively) was carried out simultaneously. The presence of cyanobacteria lysate did not affect the adsorption efficiency of these toxins to P1-CMK3 and P9-MAC particles, since no statistically significant differences were detected between adsorptions to extract and Milli-Q water control (Figure 5).

### 2.3. Effect on Cell Culture Viability of Water Exposed to Nanostructured Particles

**Figure 6:**
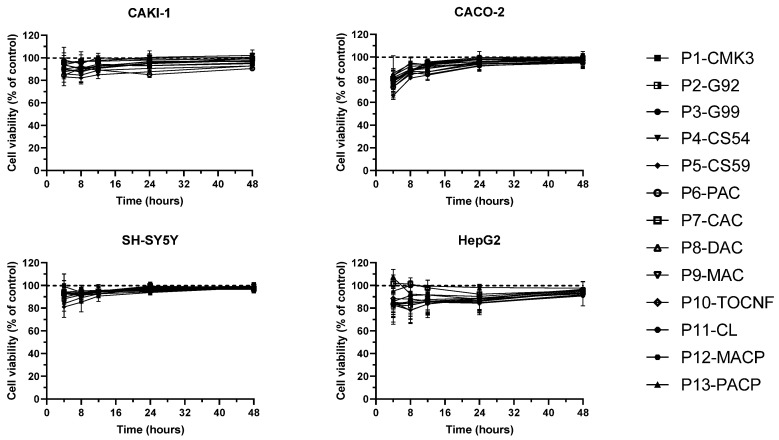
Evaluation of in vitro toxicity of particle-exposed water. Three particles of each type were incubated with 4.5 mL of Milli-Q water for 120 min at 21 °C with constant shaking; subsequently, 100 µL of particle-exposed water was added to cells cultured in 96-well plates. Cell viability was measured using the AlamarBlue viability assay. Each sample was tested in triplicate. Fluorescence measurements were carried out at 4, 8, 12, 24, and 48 h after sample addition and the results are expressed as a percentage of Milli-Q water control signal (mean ± SD, n = 3).

To ensure that the water exposed to these adsorbent nanostructured magnetic particles is harmless to humans, animals, and the environment, preliminary in vitro toxicity tests were carried out. Milli-Q water in contact with nanostructured particles for 120 min at 21 °C with constant shaking was added to cell cultures. Four human cell lines were selected for the evaluation of cell viability in the presence of these water samples: renal proximal tubule cells CAKI-1, neuroblastoma cells SH-SY5Y, large intestinal cells CACO-2, and liver cells HepG2. Cell number per well was optimised for each line to improve the detection of toxic effects within the first 24 h. Cytotoxicity was evaluated using the fluorescent cell viability test AlamarBlue by incubation of the cell culture with particle-exposed water samples at a final 50% (*v*/*v*) concentration. Fluorescence measurements were performed at 4, 8, 12, 24, and 48 h after the addition of water samples to the wells. The results are expressed as a percentage of viable cells in the testing condition, considering control cells incubated with water that was not exposed to particles as 100% viability reference. In these experiments, samples of water exposed to adsorbent particles did not alter cell viability in any of the four cell types (Figure 6). Although there seems to be a slight reduction of viability of CACO-2 cells at 4 h, there was no statistically significant difference between the fluorescence intensity obtained from cells incubated with water exposed to nanostructured particles and Milli-Q water controls.

## 3. Discussion

This study evaluates the effectiveness of particle composites of adsorption materials with MNPs for cyanotoxin removal from water. The particles’ dimensions and composition provide adequate dispersion in aqueous suspension and enable efficient extraction using a magnet, though other separation techniques, such as sedimentation or filtration, can be used to remove them from water. With an adequate system design, the magnetic properties of these particles offer the possibility of dynamic cycles of addition and withdrawal of particles from water in order to efficiently remove cyanotoxins, with cycling conditions that could be adapted to the contamination level. These characteristics make this technology highly suitable for integration into drinking water treatment plants and other water treatment facilities tailored for farming and agricultural purposes, with the purpose of removing free cyanotoxins in water after the withdrawal of cyanobacteria cells.

Thirteen nanostructured particle types were tested, with twelve different adsorption materials or different proportions of these materials, for the removal of the representative toxin of the three most prevalent cyanotoxin classes in freshwater. Nonetheless, the complexity and structural diversity of cyanotoxins have posed a challenge, as none of the tested particles has demonstrated full efficacy in eliminating the three cyanotoxins from water; instead, some particles were effective in removing one or two of these cyanotoxins. One of the main factors influencing the adsorption of cyanotoxins appears to be the pore size of the particles. Mesoporous carbon particles, P1-CMK3, were effective in eliminating MC-LR, probably because of their larger diameter pores, between 2 and 50 nm [48], which would favour the adsorption of high molecular weight toxins. On the other hand, the particles P6-PAC, P7-CAC, P8-DAC, and P9-MAC are composed of different activated carbon varieties, which have a higher content of micropores, with diameters below 2 nm [48]. The micropores probably play a crucial role in facilitating the retention of smaller molecular-sized toxins such as CYN and ATX-A. However, activated carbon particles vary in their ability to remove CYN and ATX-A, with Merck- and Desotec-activated carbon being more effective. Different adsorption properties among activated carbons from varied origins have been previously reported and attributed to the diversity of the source material for fabrication, each possessing unique characteristics [49].

Another plausible explanation for the adsorption results of CYN and ATX-A by activated carbon could be electrostatic charges. Under the experimental pH conditions (pH 5.7), activated carbon has a negative charge [50], whereas CYN has a neutral charge [51] and ATX-A has a positive charge [52]. Electrostatic interactions may cause the toxin to be attracted to the particle, with a more pronounced effect observed in the case of ATX-A. For MC-LR, this toxin carries a net negative charge at the pH conditions of the experiment, possibly contributing to the limited adsorption to activated carbon.

A noteworthy case is the P12-MACP nanostructured particles, sharing the same activated carbon as P9-MAC nanostructured particles (Merck-activated carbon) but with the addition of esterified pectin. The introduction of pectin led to a significant reduction in the efficacy of removal of low-molecular-weight toxins (CYN and ATX-A), while substantially enhancing the adsorption of MC-LR. A reduction of low-molecular-weight toxin adsorption was observed in P13-PACP particles, composed also of Panreac-activated carbon and pectin. The reduction of small toxin adsorption might be attributed to the occlusion of activated carbon small pores by pectin, preventing the binding of CYN and ATX-A. The enhancement in MC-LR adsorption, which is evident in P12-MACP nanostructured particles, could plausibly arise from either van der Waals forces, owing to the complex molecular structure of MC-LR featuring charged regions, or hydrogen bond interactions between hydroxyl groups of toxin and pectin. Electrostatic charges appear to be of minimal significance in this interaction, as MC-LR and the polysaccharide possess negative charges [53].

In addition to activated carbon composites, the TOCNF nanostructured particle, P10-TOCNF, exhibited notable adsorption of ATX-A, while graphene nanostructured particles, P2-G92 and P3-G99, showed fair adsorption. Electrostatic charge is an important factor in this scenario. The oxidation process applied to the cellulosic material of P10-TOCNF provides a negative charge to the particle [54], increasing its attraction to the positively charged ATX-A [52], which probably contributes to the remarkable adsorption values observed for these particles. However, it is worth noting that the hydrophilicity induced by this oxidation could also play a role in material adsorption [54]. Conversely, graphene maintains a neutral charge at the pH of the experiments [55], which suggests an absence of repulsive forces between the toxin and the adsorbent that may account for its moderate efficacy in ATX-A removal when compared with composites with attractive forces.

The particles with the best rates of toxin removal are P1-CMK3 for MC-LR, with a ratio of toxin adsorption per weight of 6.75 μg/g, and P9-MAC for CYN and ATX-A, with ratios of 8.08 and 3.28 μg/g, respectively. Toxin adsorption to these two particles is not affected by the presence of other cyanobacteria metabolites in the same aqueous solution, which might be released simultaneously to cyanotoxins owed to cell lysis caused by environmental factors or water treatment processes. Additionally, a high percentage of toxin was recovered after desorption of the three toxins from toxin-exposed particles, demonstrating that most of the toxin loss during the adsorption process is due to binding to the particles, and suggesting that no degradation of the toxin occurs. However, desorption protocols would have to be optimised to improve toxin recoveries in the future.

When compared to other toxin adsorption studies, the adsorption capacity achieved with these particles generally appears to be lower. However, several factors affect adsorption capacity measurements. Primarily, the inclusion of a magnetic component in the composite adds weight, due to the magnetite’s high density, without contribution to toxin adsorption. This observation is evident when comparing particles with and without a magnetic core. For instance, particles lacking a magnetic component demonstrate enhanced adsorption rates for MC-LR, when compared to adsorption studies utilising magnetic particles. Actually, adsorption for non-magnetic particles was 282 μg/g for titanium dioxide nanoparticles [56], 150 μg/g for iron oxide microparticles [57], and 3640 μg/g for polymer particles [43], while rates for magnetic particles were 160 μg/g for magnetic mesoporous silica nanospheres [58] or 168 μg/g for carbon magnetic nanoparticles [47]. Despite this, the presence of a magnetic core proves practical for the removal of particles, particularly when integrated into a real water treatment system. Another significant factor influencing adsorption is particle size, with smaller particles offering increased bonding surfaces and pore accessibility [59,60]. In contrast to the particles tested in this study, which are comparatively large (with diameters in the range of 1.4–3 mm), the existing literature reports particles with dimensions ranging from 10 to 100 nm [42], 400 to 500 nm [56], 500 nm [58], 700 nm [47], and 50 to 70 µm [57] with better adsorption. However, reducing particle size diminishes magnetic capacity [61], making extraction from water more challenging. In addition, both the incubation time and the initial toxin concentration play crucial roles. Higher concentrations generally lead to a higher amount of toxin adsorbed per gram of material, since more toxin is available to be adsorbed. Furthermore, higher incubation times increase the possibility of particle saturation. In this study, a moderately high initial toxin concentration was chosen in the range of reported concentrations in natural water conditions, considering that although MC concentrations can reach values higher than 100 µg/L, they rarely exceed 10–20 µg/L [13]. Additionally, practical incubation time of 120 min was chosen to be consistent with treatment plant operations. Previous studies have employed a wide range of initial concentrations, from 250 µg MC-LR/L [57] to 5 µg/L [56], and varied incubation times, from 48 h [56] to 5 min [58]. The diversity of these variables complicates the correlation of effectiveness with these factors.

While most publications aiming at the removal of freshwater toxins focus on the most common toxin, MC-LR, several studies have also investigated CYN and ATX-A adsorption. The factors influencing the adsorption of these toxins appear to be similar to those mentioned above for MC-LR. These studies reported adsorption ratios of 32.4 μg CYN/g and 6.8 μg ATX-A/g for magnetic carbon nanoparticles [47] or an adsorption capacity of 272.86 μg CYN/mg for magnetic polypyrrole particles [42]. In addition to particle size, other experimental conditions for these toxins that differ from our work may affect efficiency differences. As previously discussed for MC-LR, the particle size in these studies is in the nanometre range, which complicates particle extraction from the water.

None of the studies reported in the bibliography have evaluated the potential toxicity to humans or the environment of water in contact with toxin removal materials, which is crucial, as compounds or components might be released during the detoxification process that could be harmful to humans or the environment. Consequently, this study is emerging as one of the first to address the potential toxicity of toxin removal composites as a final product. The results indicate that water in contact with the particles for 2 h does not exhibit toxicity towards kidney, neuronal, liver, or intestinal cells. These cell types were selected as more susceptible to toxic effects due to the increased probability of exposure after oral intoxication through drinking water and critical physiological functions. However, it has been demonstrated that the current chemical agents utilised in water treatment processes can generate carcinogenic substances, such as trihalomethanes [35]. Therefore, the introduction of this technology into water treatment plants during toxic blooms would lead to a reduction in the usage of these chemical agents, whose concentration must be substantially increased for efficient toxin elimination [32], resulting in improved drinking water safety.

## 4. Conclusions

This study introduces a practical and effective method for removing cyanotoxins from water using magnetic nanostructured particles. Thirteen particle types were examined, and particularly those incorporating activated carbon (P9-MAC) and mesoporous carbon (P1-CMK3) on their surface exhibited the most favourable outcomes. P9-MAC particles demonstrated a remarkable 99% elimination of ATX-A and 43% of CYN, while P1-CMK3 achieved a 57% reduction in MC-LR within a solution of contaminated water at a toxic concentration. Importantly, in vitro viability experiments conducted on kidney, liver, intestinal, and neuronal cells revealed the post-treatment water not to be toxic. These findings suggest the potential of this method for effective and safe cyanotoxin elimination. However, further studies are needed to assess their effectiveness in different water scenarios, considering variations in pH, temperature, or organic matter concentration, as well as their in vivo toxicological effects.

## 5. Materials and Methods

### 5.1. Chemicals and Reagents

Acetonitrile (AcN) and methanol (MeOH), UHPLC quality, were supplied by Panreac Quimica S.A. (Barcelona, Spain). Formic acid was from Merck (Madrid, Spain). Water was purified using a Millipore Milli-Q Plus system (Millipore, Bedford, MA, USA). Durapore centrifugal filters ultrafree-MC (0.22 µm pore size) were from Millipore and nylon Spin-X^®^ centrifuge tube filters (0.22 µm pore size) were from Corning (New York, NY, USA). Certified reference materials of MC-LR, CYN, and ATX-A were provided by Cifga S.A. (Lugo, Spain). Analytical grade materials of MC-LR and CYN were provided by Enzo Biochem (New York, NY, USA). Both analytical materials were dissolved in water at a concentration of 100 µg/mL.

All in vitro culture media used, phosphate-buffered saline (PBS), foetal bovine serum (FBS), non-essential amino acids solution, trypsin–EDTA solution, and penicillin/streptomycin solution with 5000 units/mL and 5 mg/mL, respectively, were from Gibco (New York, NY, USA). Culture flasks and 96-well white, clear-bottom microplates were from Corning (New York, NY, USA).

For the nanostructured particle preparation, all chemicals were of analytical grade and used without purification. Ferric chloride (FeCl_3_·6H_2_O) was obtained from Alfa Aesar (Madrid, Spain), surfactant Tween 20 from Fluka (Steinheim, Germany), commercial activated carbon powder (MW = 12.01 g) was obtained from PANREAC (Madrid, Spain), and ferrous sulfate (FeSO_4_·7H_2_O), calcium chloride (CaCl_2_), ammonium hydroxide (NH_4_OH, 28%), and sodium alginate were purchased from Sigma-Aldrich (Saint Louis, MO, USA).

Low-molecular-weight chitosan (MW: ~50.000) was from Glentham Life Sciences (Corsham, UK), powder extra pure activated carbon from Merck (Darmstadt, Germany), powder activated carbon organosorb 200-1 WB sample from Desotec (Roeselare, Belgium), carbon black sample from Cabot Corporation (Alpharetta, GA, USA), graphene 92 and graphene 99 samples from GreenTech (Navarra, Spain), kraft lignin Lineo classic sample from Stora Enso (Kotka, Finland), and esterified pectin (esterification degree, 67 to 71%) from Acros Organics (Geel, Belgium).

### 5.2. Adsorbent Magnetic Nanostructured Particles

Magnetic nanostructured particles were synthesised via the extrusion method, incorporating magnetite (Fe_3_O_4_), iron oxyhydroxide (FeOOH), or cobalt ferrite (CoFe_2_O_4_) NPs with sodium alginate and other adsorptive components, using CaCl_2_ as the cross-linking agent.

Fe_3_O_4_ NPs were synthesised by reverse coprecipitation method [62]: CoFe_2_O_4_ NPs by the coprecipitation method using the same procedure described elsewhere [63], and FeOOH NPs via a direct hydrolysis method [64]. The NPs were cleaned and dispersed in Milli-Q water until use.

For the preparation of the magnetic nanostructured particles, a specific quantity of NPs (see Table 1) and adsorptive components were mixed under mechanical agitation at room temperature for approximately 4 h. The resulting magnetic solutions were then slowly added dropwise using a flux velocity of 1.5 mL/min into a coagulation bath containing 100 mL of 0.13 M CaCl_2_ and 450 µL Tween 20 under continuous magnetic agitation at 500 rpm using a New Era NE-300 syringe pump. Upon this addition, magnetic particles instantly formed and were left in the coagulation bath for 20 min to harden. Afterwards, the particles were cleaned with distilled water and stored in refrigerated Milli-Q water.

Morphology, structure, texture, and magnetic properties of nanostructured magnetic composites were studied in a previous work [65], where specific surface area and pore distribution were also explored for activated carbon composites. In addition, the particles demonstrated acceptable stability through several adsorption/desorption cycles of other contaminants.

### 5.3. Adsorption Experiments

Adsorption effectiveness was assessed after incubation of 3 nanostructured particles with 4.5 mL of cyanotoxin solution in Milli-Q water (pH 5.7) in a glass tube with constant shaking at an average temperature of 21 °C. MC-LR, CYN, and ATX-A were tested separately. Toxin concentrations were 60 μg/L for MC-LR and CYN and 10 μg/L for ATX-A. Samples (120 μL) were collected before and 10, 30, 60, and 120 min after adding the nanostructured particles. Adsorption efficacy was evaluated through toxin quantification using ultra-high-performance liquid chromatography coupled with tandem mass spectrometry (UHPLC-MS/MS). For each condition, at least 3 independent experiments were performed.

Adsorption experiments on cyanobacterial extracts were carried out using only the most promising particles. A total of 15 mg of a lyophilised *Aphanizomenon ovalisporum* sample (kindly provided by CIIMAR, Matosinhos, Portugal) was sonicated in 5 mL of Milli-Q water for 9 cycles, each consisting of a 1 min sonication pulse at 95% amplitude followed by a pause of 30 s. After sonication, the sample was centrifuged at 5000× *g* for 4 min at 4 °C. The pellet was resuspended with 5 mL of water and the process was repeated 3 times. Supernatants were pulled together and stored at 4 °C. A 1:15 dilution of the extract in Milli-Q water was then prepared for the adsorption experiments. Throughout the adsorption experiment, a control of Milli-Q water contaminated with the same concentration of toxins present in the diluted extract was run in parallel to the cyanobacteria lysate.

Desorption experiments were performed by washing the particles three times with Milli-Q water, followed by the addition of 2.25 mL of 75% acetonitrile. After shaking for 60 min at 21 °C, a sample of the desorption solution was evaporated, resuspended in the same volume of Milli-Q water, and quantified by UHPLC-MS/MS.

### 5.4. Cyanotoxin Quantification by Liquid Chromatography Coupled to Mass Spectrometry

Cyanotoxins present in water samples were quantified using a 1290 Infinity UHPLC system coupled to an Agilent G6460C Triple Quadrupole mass spectrometer equipped with an Agilent Jet Stream ESI source (Agilent Technologies, Waldbronn, Germany).

Before conducting the analysis, samples were filtered using nylon centrifuge filters for ATX-A and CYN, and PVDF centrifuge filters for samples containing MC-LR. Different filters were used due to the retention of ATX-A by PVDF filters and of MC-LR by nylon filters. After filtration, the samples were placed in the UHPLC autosampler at a temperature of 4 °C. Usually, detection was performed immediately after the experiment but, if required, samples were stored at −20 °C. The sample injection volume was 5 μL. Subsequently, liquid chromatography separation was performed using an ACQUITY UPLC HSS T3 column (2.1 mm × 100 mm, 1.8 µm, Waters) at 35 °C. The mobile phases consisted of water (A) and AcN (B), both containing 0.05% formic acid, with a flow rate of 0.4 mL/min. For CYN and ATX-A, the gradient of mobile phase B was as follows: 2% for 4 min, linear increase to 70% over 4 min, 70% for 1 min, linear decrease to 2% over 1 min, and final re-equilibration at 2% for 3 min. For MC-LR, the gradient of mobile phase B was as follows: 2% for 4 min, linear increase to 85% over 4.5 min, 85% for 4 min, linear decrease to 2% over 1 min, and final re-equilibration at 2% for 4 min.

Regarding mass spectrometry, the optimised electrospray ionisation (ESI) source parameters were as follows: sheath gas flow rates of 12 L/min for MC-LR and 11 L/min for CYN and ATX-A; gas temperature of 280 °C for MC-LR and 250 °C for CYN and ATX-A; gas flow rate of 8 L/min; nebulizer pressure of 30 psi; capillary voltage for positive ionisation of 3500 V for MC-LR and 3000 V for CYN and ATX-A; and nozzle voltages for positive ionisation of 1500 V for MC-LR and 1000 V for CYN and ATX-A. MRM acquisition mode was used for toxin identification and quantification. For MC-LR, the quantification transition was 498.28 > 135 (collision energy (CE) and fragmentor were 8 and 105, respectively); confirmatory transitions were 995.56 > 135 (CE of 80 and fragmentor of 220) and 498.28 > 861.4 (CE of 8 and fragmentor of 105). For CYN, quantification was carried out with transition 416.13 > 194.2 and confirmation with 416.13 > 336.1 (CE and fragmentor parameters were 36 and 160, respectively). Regarding ATX-A, 166.13 > 43.2 was used as a quantification transition, and 166.13 > 91.1 was used for confirmation (CE and fragmentor values were 28 and 90, respectively). Positive polarity was used for all molecules and the collision-activated voltage (CAV) was set at 1.

Duplicate calibration curves were generated for each analysis, covering concentrations of MC-LR and CYN ranging from 0.25 to 128 µg/L, and ATX-A ranging from 0.05 to 30 µg/L. Each sample was quantified twice; the results of each condition in one experiment are the average of both measurements.

### 5.5. Cell Line Cultures

Four cell lines were used to evaluate the in vitro toxicity of these materials: proximal tubular renal cells CAKI-1 at passages 8–18, neuroblastoma cells SH-SY5Y at passages 30–40, liver cells HepG2 at passages 3–10, and large intestine cells CACO-2 at passages 25–35. Media used to maintain the growth of cell cultures were RPMI 1640, DMEM/F12, and MEM supplemented with 10% FBS and 1% penicillin/streptomycin for CAKI-1, SH-SY5Y, and HepG2, respectively. For CACO-2, MEM medium supplemented with 10% FBS, 1% penicillin/streptomycin, and 1% non-essential amino acids was utilised. All cell lines were cultured in 75 cm^2^ flasks and subcultured when the cell confluence reached 80%.

### 5.6. Cytotoxicity Evaluation in Cell Cultures

Water samples exposed for 2 h to the nanostructured particles in the absence of toxins were pipetted from experiments performed under the previously described conditions (4.5 mL of Milli-Q water, 3 particles, constant shaking). Experiments for cytotoxicity evaluation were conducted in 96-well white, clear-bottom microplates. AlamarBlue cytotoxicity assay (ThermoFisher, Waltham, MA, USA) was employed as viability reporter. Prior to conducting the experiments, cells were collected from maintenance flasks, transferred in 80 µL to plate wells, and allowed to attach to the well bottom for 24 h. Cell concentrations per well were 1500 cells for CAKI-1, 8000 cells for SH-SY5Y, 2500 cells for HepG2, and 5000 cells for CACO-2. Then, the sample and AlamarBlue were added to the plate well with a final concentration of 50% and 10% (*v*/*v*), respectively. For each experiment, a positive viability control using Milli-Q water and a negative viability control employing saponin at a concentration of 1 mg/mL were conducted. Fluorescence generated following AlamarBlue reduction by viable cell metabolism was measured at 4, 8, 12, 24, and 48 h using a plate reader (Synergy, Biotek, Winooski, VT, USA) with excitation and emission wavelengths of 530 nm and 590 nm, respectively.

### 5.7. Statistical Analysis

All data are expressed as mean ± SD and were obtained from at least 3 independent experiments performed in duplicates for adsorption assays and in triplicates for cell viability assays. Statistical comparison was conducted by one-way ANOVA, utilising GraphPad Prism 9 (GraphPad Software, Inc., La Jolla, CA, USA). *p* values < 0.05 were considered statistically significant.

## Figures and Tables

**Table 1:** Magnetic nanostructured particles with adsorbent coating tested in this study. Dry weight, diameter, and composition per particle are shown. Composition is reported as the percent of each component versus total weight.

Particle Code	Weight (mg/Particle)	Diameter (cm)	Composition (Wt. %)
P1-CMK3	6.1	0.20	Fe_3_O_4_ (17.9%); sodium alginate (51.3%) and CMK-3 mesoporous carbon (30.8%).
P2-G92	4.3	0.20	Fe_3_O_4_ (20.6%); sodium alginate (45.4%) and graphene 92 (34.0%).
P3-G99	6.9	0.21	Fe_3_O_4_ (23.1%); sodium alginate (43.9%) and graphene 99 (33%).
P4-CS54	3.3	0.16	FeO(OH) (45.2%) and chitosan (54.8%).
P5-CS59	2.9	0.15	FeO(OH) (40.3%) and chitosan (59.7%).
P6-PAC	3.4	0.30	Fe_3_O_4_ (13.3%); sodium alginate (34.7%) and Panreac-activated carbon (52%).
P7-CAC	2.3	0.15	Fe_3_O_4_ (18.3%); Cabot-activated carbon (16.3%) and sodium alginate (65.4%).
P8-DAC	4.5	0.22	Fe_3_O_4_ (10.9%); Desotec-activated carbon (53.5%) and sodium alginate (35.6%).
P9-MAC	6.0	0.30	Fe_3_O_4_ (7.92%); Merck-activated carbon (55.2%) and sodium alginate (36.8%).
P10-TOCNF	3.2	0.15	Fe_3_O_4_ (3.78%); sodium alginate (3.78%); CMK-3 mesoporous carbon (0.4%) and TOCNF * (92%).
P11-CL	2.4	0.14	CoFe_2_O_4_ (9.8%); sodium alginate (81.1%) and calcined lignin (9.1%).
P12-MACP	2.2	0.15	Fe_3_O_4_ (8.4%); Merck-activated carbon (30.5%); sodium alginate (36.7%) and esterified pectin (24.4%).
P13-PACP	3.2	0.20	Fe_3_O_4_ (8.76%); Panreac-activated carbon (30.41%); sodium alginate (36.5%) and esterified pectin (24.33%).

* 2,2,6,6-tetramethylpiperidine-1-oxyl (TEMPO)-oxidised cellulose nanofibers (TOCNF).

## Data Availability

Dataset available on request from the authors.

## References

[1] Buratti F.M., Manganelli M., Vichi S., Stefanelli M., Scardala S., Testai E., Funari E. (2017). Cyanotoxins: Producing organisms, occurrence, toxicity, mechanism of action and human health toxicological risk evaluation. Arch. Toxicol..

[2] Chorus I., Fastner J., Welker M. (2021). Cyanobacteria and cyanotoxins in a changing environment: Concepts, controversies, challenges. Water.

[3] Meriluoto J., Blaha L., Bojadzija G., Bormans M., Brient L., Codd G.A., Drobac D., Faassen E.J., Fastner J., Hiskia A. (2017). Toxic cyanobacteria and cyanotoxins in European waters–recent progress achieved through the CYANOCOST Action and challenges for further research. Adv. Oceanogr. Limnol..

[4] Hauer T., Mühlsteinová R., Bohunická M., Kaštovský J., Mareš J. (2015). Diversity of cyanobacteria on rock surfaces. Biodivers. Conserv..

[5] Whitton B.A., Potts M., Whitton B.A. (2012). Introduction to the Cyanobacteria. Ecology of Cyanobacteria II: Their Diversity in Space and Time.

[6] Holland A., Kinnear S. (2013). Interpreting the possible ecological role (s) of cyanotoxins: Compounds for competitive advantage and/or physiological aide?. Mar. Drugs.

[7] Merel S., Walker D., Chicana R., Snyder S., Baurès E., Thomas O. (2013). State of knowledge and concerns on cyanobacterial blooms and cyanotoxins. Environ. Int..

[8] Moreira C., Vasconcelos V., Antunes A. (2022). Cyanobacterial blooms: Current knowledge and new perspectives. Earth.

[9] Lürling M., Van Oosterhout F., Faassen E. (2017). Eutrophication and warming boost cyanobacterial biomass and microcystins. Toxins.

[10] O’Neil J.M., Davis T.W., Burford M.A., Gobler C.J. (2012). The rise of harmful cyanobacteria blooms: The potential roles of eutrophication and climate change. Harmful Algae.

[11] Churro C., Dias E., Valério E. (2012). Risk assessment of cyanobacteria and cyanotoxins, the particularities and challenges of Planktothrix spp. Monitoring. Novel Approaches and Their Applications in Risk Assessment.

[12] WHO (2011). Guidelines for Drinking-Water Quality.

[13] WHO (2020). Cyanobacterial Toxins: Microcystins.

[14] WHO (2020). Cyanobacterial Toxins: Cylindrospermopsins.

[15] WHO (2020). Cyanobacterial Toxins: Anatoxin-a and Analogues.

[16] Svirčev Z., Lalić D., Bojadžija Savić G., Tokodi N., Drobac Backović D., Chen L., Meriluoto J., Codd G.A. (2019). Global geographical and historical overview of cyanotoxin distribution and cyanobacterial poisonings. Arch. Toxicol..

[17] Biré R., Bertin T., Dom I., Hort V., Schmitt C., Diogène J., Lemée R., De Haro L., Nicolas M. (2020). First evidence of the presence of anatoxin-a in sea figs associated with human food poisonings in France. Mar. Drugs.

[18] Drobac D., Tokodi N., Simeunović J., Baltić V., Stanić D., Svirčev Z. (2013). Human exposure to cyanotoxins and their effects on health. Arh. Za Hig. Rada I Toksikol..

[19] Giannuzzi L., Sedan D., Echenique R., Andrinolo D. (2011). An acute case of intoxication with cyanobacteria and cyanotoxins in recreational water in Salto Grande Dam, Argentina. Mar. Drugs.

[20] Alosman M., Cao L., Massey I.Y., Yang F. (2021). The lethal effects and determinants of microcystin-LR on heart: A mini review. Toxin Rev..

[21] Arman T., Baron J.A., Lynch K.D., White L.A., Aldan J., Clarke J.D. (2021). MCLR-elicited hepatic fibrosis and carcinogenic gene expression changes persist in rats with diet-induced nonalcoholic steatohepatitis through a 4-week recovery period. Toxicology.

[22] Arman T., Lynch K.D., Montonye M.L., Goedken M., Clarke J.D. (2019). Sub-chronic microcystin-LR liver toxicity in preexisting diet-induced nonalcoholic steatohepatitis in rats. Toxins.

[23] He J., Li G., Chen J., Lin J., Zeng C., Chen J., Deng J., Xie P. (2017). Prolonged exposure to low-dose microcystin induces nonalcoholic steatohepatitis in mice: A systems toxicology study. Arch. Toxicol..

[24] IARC (2010). Ingested Nitrate and Nitrite, and Cyanobacterial Peptide Toxins. IARC Monographs on the Evaluation of Carcinogenic Risks to Humans.

[25] Falconer I.R., Humpage A.R. (2006). Cyanobacterial (blue-green algal) toxins in water supplies: Cylindrospermopsins. Environ. Toxicol. Int. J..

[26] Pouria S., de Andrade A., Barbosa J., Cavalcanti R., Barreto V., Ward C., Preiser W., Poon G.K., Neild G., Codd G. (1998). Fatal microcystin intoxication in haemodialysis unit in Caruaru, Brazil. Lancet.

[27] Fastner J., Beulker C., Geiser B., Hoffmann A., Kröger R., Teske K., Hoppe J., Mundhenk L., Neurath H., Sagebiel D. (2018). Fatal neurotoxicosis in dogs associated with tychoplanktic, anatoxin-a producing *Tychonema sp.* in mesotrophic lake Tegel, Berlin. Toxins.

[28] Saker M., Thomas A., Norton J. (1999). Cattle mortality attributed to the toxic cyanobacterium *Cylindrospermopsis raciborskii* in an outback region of north Queensland. Environ. Toxicol. Int. J..

[29] Svirčev Z., Drobac D., Tokodi N., Mijović B., Codd G.A., Meriluoto J. (2017). Toxicology of microcystins with reference to cases of human intoxications and epidemiological investigations of exposures to cyanobacteria and cyanotoxins. Arch. Toxicol..

[30] Lawton L.A., Robertson P.K. (1999). Physico-chemical treatment methods for the removal of microcystins (cyanobacterial hepatotoxins) from potable waters. Chem. Soc. Rev..

[31] Nicholson B.C., Rositano J., Burch M.D. (1994). Destruction of cyanobacterial peptide hepatotoxins by chlorine and chloramine. Water Res..

[32] Rodríguez E., Onstad G.D., Kull T.P., Metcalf J.S., Acero J.L., von Gunten U. (2007). Oxidative elimination of cyanotoxins: Comparison of ozone, chlorine, chlorine dioxide and permanganate. Water Res..

[33] Vlad S., Anderson W.B., Peldszus S., Huck P.M. (2014). Removal of the cyanotoxin anatoxin-a by drinking water treatment processes: A review. J. Water Health.

[34] Yan S., Jia A., Merel S., Snyder S.A., O’Shea K.E., Dionysiou D.D., Song W. (2016). Ozonation of cylindrospermopsin (cyanotoxin): Degradation mechanisms and cytotoxicity assessments. Environ. Sci. Technol..

[35] Wang G.-S., Deng Y.-C., Lin T.-F. (2007). Cancer risk assessment from trihalomethanes in drinking water. Sci. Total Environ..

[36] Zhang X., Li J., Yang J.-Y., Wood K.V., Rothwell A.P., Li W., Blatchley III E.R. (2016). Chlorine/UV process for decomposition and detoxification of microcystin-LR. Environ. Sci. Technol..

[37] Lemes G.A., Kersanach R., Pinto L.d.S., Dellagostin O.A., Yunes J.S., Matthiensen A. (2008). Biodegradation of microcystins by aquatic *Burkholderia sp.* from a South Brazilian coastal lagoon. Ecotoxicol. Environ. Saf..

[38] Toporowska M. (2022). Degradation of three microcystin variants in the presence of the macrophyte *Spirodela polyrhiza* and the associated microbial communities. Int. J. Environ. Res. Public Health.

[39] Yang F., Huang F., Feng H., Wei J., Massey I.Y., Liang G., Zhang F., Yin L., Kacew S., Zhang X. (2020). A complete route for biodegradation of potentially carcinogenic cyanotoxin microcystin-LR in a novel indigenous bacterium. Water Res..

[40] Kumar P., Pérez J.A.E., Cledon M., Brar S.K., Duy S.V., Sauvé S., Knystautas É. (2020). Removal of microcystin-LR and other water pollutants using sand coated with bio-optimized carbon submicron particles: Graphene oxide and reduced graphene oxide. Chem. Eng. J..

[41] Pavagadhi S., Tang A.L.L., Sathishkumar M., Loh K.P., Balasubramanian R. (2013). Removal of microcystin-LR and microcystin-RR by graphene oxide: Adsorption and kinetic experiments. Water Res..

[42] Hena S., Rozi R., Tabassum S., Huda A. (2016). Simultaneous removal of potent cyanotoxins from water using magnetophoretic nanoparticle of polypyrrole: Adsorption kinetic and isotherm study. Environ. Sci. Pollut. Res..

[43] Krupadam R.J., Patel G.P., Balasubramanian R. (2012). Removal of cyanotoxins from surface water resources using reusable molecularly imprinted polymer adsorbents. Environ. Sci. Pollut. Res..

[44] Kim S., Yun Y.-S., Choi Y.-E. (2018). Development of waste biomass based sorbent for removal of cyanotoxin microcystin-LR from aqueous phases. Bioresour. Technol..

[45] Huang C., Zhang W., Yan Z., Gao J., Liu W., Tong P., Zhang L. (2015). Protonated mesoporous graphitic carbon nitride for rapid and highly efficient removal of microcystins. RSC Adv..

[46] Li L., Qiu Y., Huang J., Li F., Sheng G.D. (2014). Mechanisms and factors influencing adsorption of microcystin-LR on biochars. Water Air Soil Pollut..

[47] González-Jartín J.M., de Castro Alves L., Alfonso A., Piñeiro Y., Vilar S.Y., Rodríguez I., Gomez M.G., Osorio Z.V., Sainz M.J., Vieytes M.R. (2020). Magnetic nanostructures for marine and freshwater toxins removal. Chemosphere.

[48] Storck S., Bretinger H., Maier W.F. (1998). Characterization of micro-and mesoporous solids by physisorption methods and pore-size analysis. Appl. Catal. A Gen..

[49] Abbas T., Kajjumba G.W., Ejjada M., Masrura S.U., Marti E.J., Khan E., Jones-Lepp T.L. (2020). Recent advancements in the removal of cyanotoxins from water using conventional and modified adsorbents—A contemporary review. Water.

[50] Huang W.-J., Cheng B.-L., Cheng Y.-L. (2007). Adsorption of microcystin-LR by three types of activated carbon. J. Hazard. Mater..

[51] Ho L., Lambling P., Bustamante H., Duker P., Newcombe G. (2011). Application of powdered activated carbon for the adsorption of cylindrospermopsin and microcystin toxins from drinking water supplies. Water Res..

[52] Klitzke S., Beusch C., Fastner J. (2011). Sorption of the cyanobacterial toxins cylindrospermopsin and anatoxin-a to sediments. Water Res..

[53] Li J., Yang Z.-L., Ding T., Song Y.-J., Li H.-C., Li D.-Q., Chen S., Xu F. (2022). The role of surface functional groups of pectin and pectin-based materials on the adsorption of heavy metal ions and dyes. Carbohydr. Polym..

[54] Wei J., Chen Y., Liu H., Du C., Yu H., Ru J., Zhou Z. (2016). Effect of surface charge content in the TEMPO-oxidized cellulose nanofibers on morphologies and properties of poly (N-isopropylacrylamide)-based composite hydrogels. Ind. Crops Prod..

[55] Kartick B., Srivastava S. (2013). Green synthesis of graphene. J. Nanosci. Nanotechnol..

[56] Okupnik A., Contardo-Jara V., Pflugmacher S. (2015). Potential role of engineered nanoparticles as contaminant carriers in aquatic ecosystems: Estimating sorption processes of the cyanobacterial toxin microcystin-LR by TiO_2_ nanoparticles. Colloids Surf. A: Physicochem. Eng. Asp..

[57] Gao Y.Q., Gao N.Y., Deng Y., Gu J.S., Shen Y.C., Wang S.X. (2012). Adsorption of microcystin-LR from water with iron oxide nanoparticles. Water Environ. Res..

[58] Deng Y., Qi D., Deng C., Zhang X., Zhao D. (2008). Superparamagnetic high-magnetization microspheres with an Fe_3_O_4_@ SiO_2_ core and perpendicularly aligned mesoporous SiO_2_ shell for removal of microcystins. J. Am. Chem. Soc..

[59] Preethi S., Sivasamy A., Sivanesan S., Ramamurthi V., Swaminathan G. (2006). Removal of safranin basic dye from aqueous solutions by adsorption onto corncob activated carbon. Ind. Eng. Chem. Res..

[60] Sun G., Shi W. (1998). Sunflower stalks as adsorbents for the removal of metal ions from wastewater. Ind. Eng. Chem. Res..

[61] Patsula V., Moskvin M., Dutz S., Horák D. (2016). Size-dependent magnetic properties of iron oxide nanoparticles. J. Phys. Chem. Solids.

[62] de Castro Alves L., Yáñez-Vilar S., Piñeiro-Redondo Y., Rivas J. (2019). Novel magnetic nanostructured beads for cadmium (II) removal. Nanomaterials.

[63] Darwish M.S., Kim H., Lee H., Ryu C., Lee J.Y., Yoon J. (2019). Synthesis of magnetic ferrite nanoparticles with high hyperthermia performance via a controlled co-precipitation method. Nanomaterials.

[64] Fan J., Zhao Z., Ding Z., Liu J. (2018). Synthesis of different crystallographic FeOOH catalysts for peroxymonosulfate activation towards organic matter degradation. RSC Adv..

[65] de Castro Alves L., Yáñez-Vilar S., Piñeiro-Redondo Y., Rivas J. (2020). Efficient separation of heavy metals by magnetic nanostructured beads. Inorganics.

